# FACE-TO-FACE AND FACEBOOK ACROSS THE LIFESPAN: SOCIAL INTERACTIONS BUFFER STRESS ON MOOD, BUT ONLY FOR ADOLESCENTS

**DOI:** 10.1093/geroni/igz038.2558

**Published:** 2019-11-08

**Authors:** Yin Liu, Elizabeth B Fauth, Myles Maxey, Troy Beckert

**Affiliations:** Utah State University, Logan, Utah, United States

## Abstract

Social support serves as a protective factor, buffering stress in both adolescents and adults, however Socioemotional Selectivity Theory suggests developmental differences in stress reactivity and social support. It is unclear how modern forms of social contact, such as social media buffer stress, and the extent to which this differs across the lifespan. We utilized ecological momentary data to examine the moderating effects of age and two distinct types of social contacts the person had experienced in prior hours (frequency of face-to-face, or social media contacts) on the association between daily stress and momentary mood. Participants were recruited initially through Amazon.com’s Mechanical Turk (adolescents referred by a parent). A total of 119 adolescent (n = 44; Agemean= 15.73) and middle-aged/older adult participants (n = 75; Agemean= 59.67) provided momentary data three times a day, on three consecutive days, every two weeks, for up to 12 weeks. Multi-level models showed significant 3-way interactions between stress appraisal of avoiding an argument, age group, and frequency of social contact via face-to-face (β = 1.698, se = 0.542, p = .002) and social media (β = 3.341, se = 0.984, p = .001). Older adults experienced better mood than adolescents. When avoiding an argument was appraised as more stressful, both age groups displayed worse mood. Whereas high levels of recent social contact (both face-to-face and social media) seemed to exacerbate the impact of this stressor on poorer mood for older persons, high levels of recent social contact, particularly social media, had stress-buffering benefits for adolescents.

